# North, North, North and the border between academia and research institutions

**DOI:** 10.1063/4.0001083

**Published:** 2025-10-27

**Authors:** Vivian Stojanoff

**Affiliations:** National Synchrotron Light Source II, Brookhaven National Laboratory, Upton, NY, USA

## Abstract

It was summer … I was freezing as I entered the Civil Engineering building of the Polytechnique School of Engineering where the entrance exam for the University of Sao Paulo (USP) Schools and Institutes was being held … Following my BSc degree in Physics, I got an MSc degree in Condensed Matter and Material Science and continued to obtain a PhD in Experimental Physics and Crystallography from the Physics Institute, USP. It was as a graduate student that I started to spin the thread that has been defining my career … The love for crystals and X-rays, and an obsession for light sources …

Today Vivian Stojanoff leads the User, Training and Outreach program of the Center for Biomolecular Structure (CBMS) at the National Synchrotron Light Source II. Her research focus on the application and development of X-ray characterization and crystallization methods of biomolecules.

